# Neurobiology of Subtypes of Trichotillomania and Skin Picking Disorder

**DOI:** 10.1017/S109285292100095X

**Published:** 2021-11-03

**Authors:** Jon E. Grant, Richard A.I. Bethlehem, Samuel R. Chamberlain, Tara S. Peris, Emily J. Ricketts, Joseph O’Neill, Darin D. Dougherty, Dan Stein, Christine Lochner, Douglas W. Woods, John Piacentini, Nancy J. Keuthen

**Affiliations:** 1 Department of Psychiatry & Behavioral Neuroscience University of Chicago, Chicago, IL, USA; 2 Department of Psychiatry, University of Cambridge, UK; 3 Department of Psychiatry, Faculty of Medicine, University of Southampton, UK; and Southern Health NHS Foundation Trust, UK; 4 Department of Psychiatry and Biobehavioral Sciences, University of California, Los Angeles, CA, USA; 5 Department of Psychiatry, Massachusetts General Hospital and Harvard Medical School, Boston, USA; 6 Department of psychiatry, University of Cape Town, South Africa; 7 Department of Psychiatry, Stellenbosch University, South Africa; 8 Department of Psychology, Marquette University, Milwaukee, WI, USA

**Keywords:** trichotillomania, skin picking disorder, imaging, subtypes, neurobiology

## Abstract

**Objective:**

Trichotillomania and skin picking disorder are common and often debilitating mental health conditions, grouped under the umbrella term of body focused repetitive behaviors (BFRBs). Recent clinical subtyping found that there were three distinct subtypes of trichotillomania and two of skin picking disorder. Whether these clinical subtypes map on to any unique neurobiological underpinnings however remains unknown.

**Methods:**

251 adults (193 with a BFRB [85.5% (n=165) female] and 58 healthy controls [77.6% (n=45) female]) were recruited from the community for a multi-center between-group comparison using structural neuroimaging. Differences in whole brain structure were compared across the subtypes of BFRBs, controlling for age, sex, scanning site and intracranial volume.

**Results:**

When the subtypes of TTM were compared, low awareness hair pullers demonstrated increased cortical volume in the lateral occipital lobe relative to controls and sensory sensitive pullers. In addition, impulsive/perfectionist hair pullers showed relative decreased volume near the lingual gyrus of the inferior occipital-parietal lobe compared to controls.

**Conclusions:**

These data indicate that the anatomical substrates of particular forms of BFRBs are dissociable, which may have implications for understanding clinical presentations and treatment response.

## Introduction

Trichotillomania (TTM) and Skin Picking Disorder (SPD), are characterized by repeated pulling out of hair resulting in hair loss or picking at skin resulting in tissue damage, respectively. These disorders have been conceptualized under the larger umbrella concept of body focused repetitive behavior disorders (BFRBs). Despite decades of research, effective treatments for BFRBs remain elusive.^1–2^ One issue that has thwarted treatment development for BFRBs is the lack of any clear pathophysiological targets (multiple brain areas and circuits have been examined in small studies and oftentimes conflicting studies^3–18^).

In the related area of obsessive compulsive disorder (OCD), recent research suggests that subtypes of OCD may in fact have partially distinct biological underpinnings. Using magnetic resonance imaging (MRI) in 37 OCD patients and 37 matched controls, Okada and colleagues found significant negative correlations between symptomatic dimension scores and regional GM volumes such as decreased right cerebellum in ‘aggression/checking’ and decreased right insula in ‘contamination/washing’.^19^ In another study, this time using a different approach to subtyping in OCD, Subira and colleagues^20^ examined the structural biology using a two-group classification of OCD, in 95 people with OCD and 95 controls. They found that in comparison to the autogenous group, reactive patients showed larger gray matter volumes in the right rolandic operculum. When compared to healthy controls, reactive patients showed larger volumes in the putamen (bilaterally), while autogenous patients showed a smaller left anterior temporal lobe. What becomes clear from this limited research is that if one is to find meaningful biological differences in subtypes of a disorder, that those subtypes should be well established.

Toward that end, we recently completed the largest and most comprehensive multi-site phenomenological study of BFRBs.^21^ Using multiple clinical and cognitive measures and advanced statistical methodology, we found evidence for three subtypes of TTM with unique clinical presentations: one subtype referred to as “sensory sensitive pullers” characterized by highly focused pulling, but infrequent and low intensity urges to pull; another referred to as “low awareness pullers” characterized by automatic pulling and pulling due to emotional triggers; and a final subtype of “impulsive/perfectionist pullers” who pull to control unpleasant feelings and feel unable to resist their pulling. In terms of SPD, we identified two potential subtypes: “emotional/reward pickers” with strong and frequent urges to pick, picking from negative emotions as well as automatic picking, and reporting little control; and a second subtype of “functional pickers” who have fairly mild SPD, lower urges to pick, and overall little distress or impact from the picking.^21^

Although our recent research allows for greater understanding of the clinical heterogeneity of these disorders, a similar understanding of the neurobiology of BFRBs remains limited. Therefore, if the clinical subtypes are potentially meaningful it would require that there be an identifiable neurobiology of the subtypes. Thus, the objective of this study was to examine brain structure across the subtypes of TTM and SPD to determine if the clinical subtyping reflected unique biological underpinnings.

## Methods

Participants included 251 adults recruited from the community and identified as having either a BFRB (meeting DSM-5 criteria for TTM, SPD, or both as their primary psychiatric problem) or being a healthy control. Four sites were involved in recruitment: University of Chicago, University of California, Los Angeles, and Massachusetts General Hospital/Harvard Medical School, and Stellenbosch University, South Africa. Recruitment started in October 2017 and ended in March 2019.

Inclusion criteria for the clinical sample were: a) DSM-5 diagnosis of TTM and/or SPD; b) aged 18 to 65 years; c) fluency in English; and d) capable of providing informed consent. Inclusion criteria for the healthy controls were the same except they could have no current or lifetime history of any DSM-5 psychiatric disorder based on screening (see below).

Exclusion criteria for the clinical sample and healthy controls were: (a) current or lifetime diagnosis of any serious medical or psychiatric illness that would preclude successful study participation, based on screening; (b) neurological conditions that would preclude completion of neurocognitive tasks; (c) use of psychotropic medications unless the dose had been stable for at least the past 3 months; (d) change in frequency or type of psychotherapy for at least the past 3 months; (e) body metal other than dental fillings (assessed using a neuroimaging screening form); (f) positive pregnancy test for females of childbearing age; and (g) medical condition or other factor that would interfere in the subject’s ability to participate in the study.

### Procedures

Potential participants were screened by the study site coordinator, who then scheduled an interview date. After receiving a complete description of the study, participants provided written informed consent. Participants received a cash incentive for participation to reimburse them for their time and transport costs. The authors assert that all procedures contributing to this work complied with the ethical standards of the relevant national and institutional committees on human experimentation and with the Helsinki Declaration of 1975, as revised in 2008. All procedures involving human subjects were approved by the Institutional Review Boards at each of the participating universities. Data sharing agreements were arranged across all sites.

### Assessments

All participants completed a comprehensive diagnostic interview (Mini International Neuropsychiatric Interview 7.0 (MINI 7.0)^22^; BFRB diagnostic modules and symptom severity scales; and self-report questionnaires regarding BFRB symptoms, general psychopathology, quality of life, and family environment. A detailed list of the assessments is provided in an earlier publication.^21^

### MRI Neuroimaging

All participants were asked to complete an “MR Screening Form” to rule out any conditions that preclude MR scanning. We used a multi-site neuroimaging design involving participants across four sites: (1) MRI Research Center at the University of Chicago; (2) Massachusetts General Hospital Martinos Center for Biomedical Imaging; (3) Staglin Center for Cognitive Neuroscience at the UCLA Semel Institute for Neuroscience and Human Behavior, and (4)The Cape Universities Body Imaging Centre, Cape Town.. As described above, participants were screened for scanner compatibility at the outset, and we scanned eligible participants sequentially. Imaging was performed on a 3-Tesla MRI scanner at all three sites with all scanners synchronized via TTL pulse. In addition, a set of parameters compatible with all scanners, particularly those directly affecting image contrast or signal-to-noise, were devised and held constant across sites. Another quality control procedure which was implemented at each site was using a phantom scan to provide information about geometric distortions and signal uniformity related to hardware differences in radiofrequency coils and gradient systems, image contrast and temporal stability. Each MRI scanning session lasted no more than 75 minutes. We first acquired high resolution, anatomical images, typically about 15 minutes.

**Figure 1:**
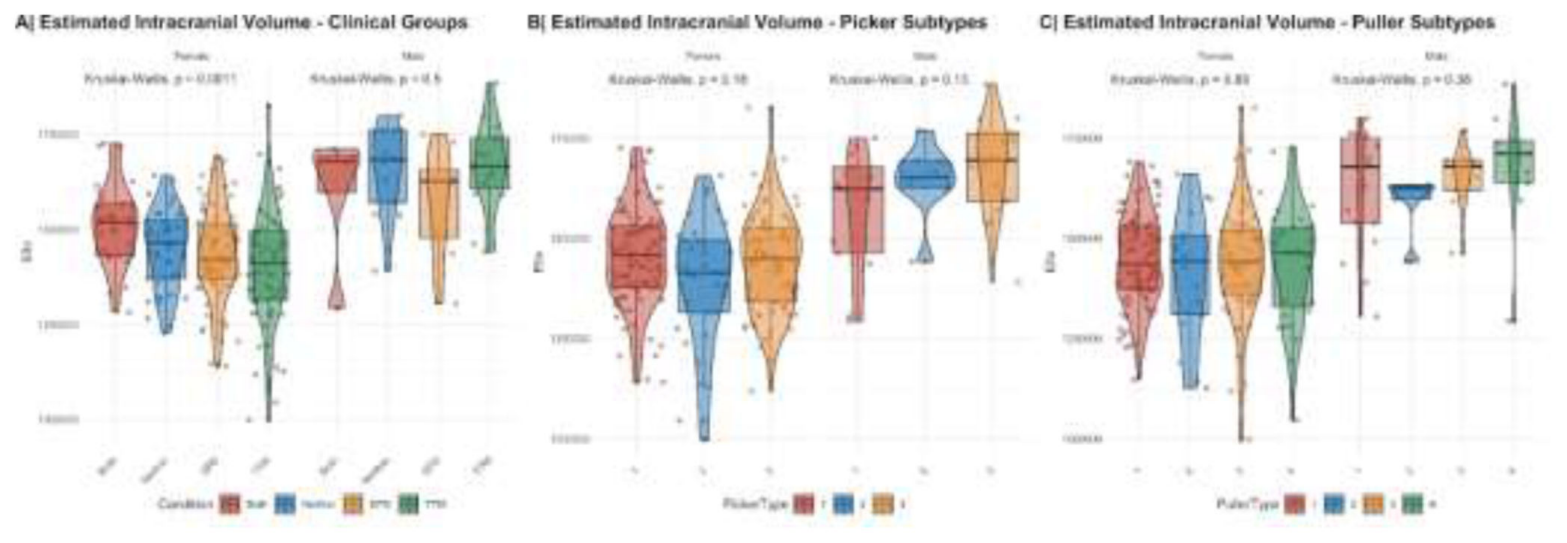


**Figure 2:**
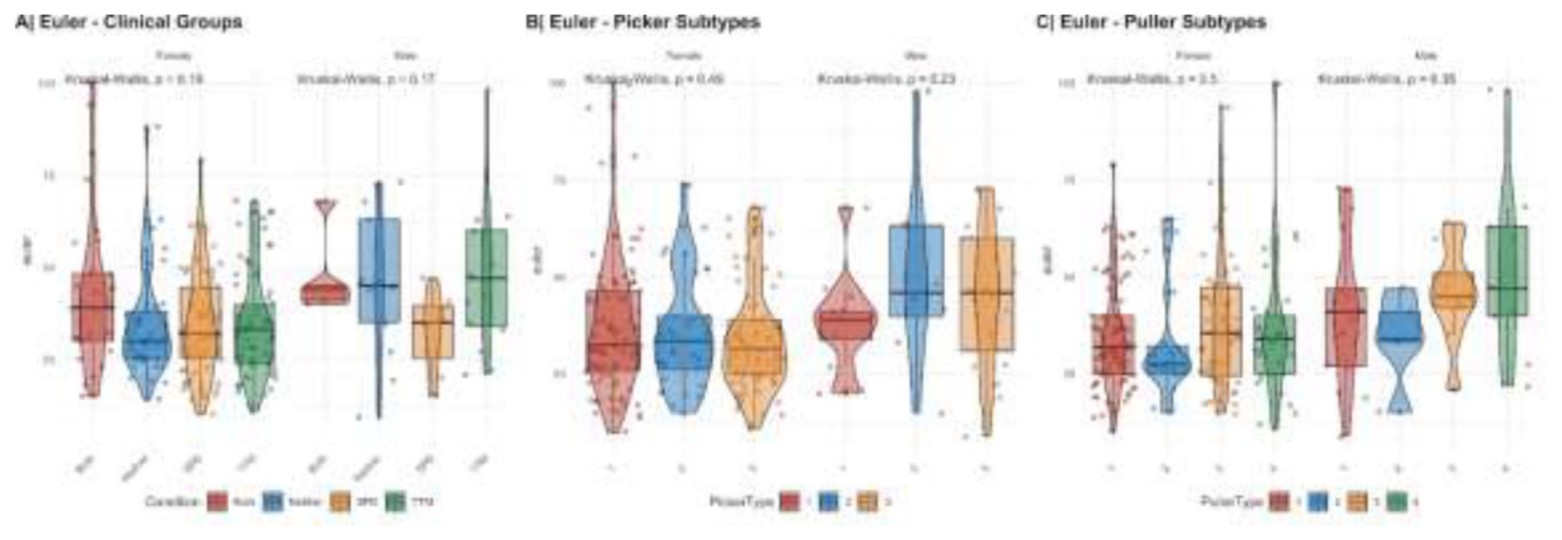


Structural T1-weighted MPRAGE data for 251 adult subjects was preprocessed with Freesurfer (v6.0.1).^23^Recon-all reconstruction included bias field correction, registration to stereotaxic space, intensity normalization, skull-stripping, and white matter segmentation. Freesurfers qcache option was used to obtain fsaverage maps at different full width at half maximum (FWHM) Gaussian smoothing kernels for post-processing and statistical analyses. Quality control to explore potential group differences in quality or known confounders included the extraction of the Euler index^24^, a proxy for the quality of the image reconstruction, and an assessment of potential group differences in total intracranial volume (Fig 1). Quality control revealed no systematic differences in reconstruction quality (Fig 2), and small but significant systematic group differences in the female clinical groups for total brain volume. Thus, any subsequent analyses included total intracranial volume as a covariate.

Subject morphometric maps for volume, thickness and surface area were all registered to fsaverage space using the mris_preproc method implemented in Freesurfer for the 3 different smoothing kernels (5, 10 and 15 mm). Previously reported subtypes on two dimensions, pickers and pullers, were analyzed using the general linear model framework implemented in Freesurfer accounting for sex, age, site and estimated intracranial volume. Subtype comparisons within each morphological feature were corrected for multiple comparisons using a cluster threshold of p < 0.001 as recommended by Greve & Fischl^25^ to avoid false positives. Only results that showed consistent effects across all three levels of smoothness were considered significant, but all results across smoothness kernels are included in the supplementary materials.

## Results

### Sample Characteristics

The sample included 251 adults (193 with a BFRB [85.5% (n=165) female] and 58 healthy controls [77.6% (n=45) female]). Based on previously conducted mixture modeling analysis, individuals were categorized as belonging to various subtypes of pulling or picking (Table 1).

### Puller subtypes

Puller subtype 3 (low awareness pullers) showed increased cortical volume in the lateral occipital lobe relative to both subtype 1 (controls) and 2 (sensory sensitive pullers). In addition, subtype 4 (impulsive/perfectionist pullers) showed relatively decreased volume near the lingual gyrus of the inferior occipital-parietal lobe compared to controls.

### Picker subtypes

No regions showed robust significant group differences at the rigorous criteria thresholds. Some regions showed small volumetric differences between group 1 (controls) and group 3 (emotional/reward pickers) but these did not retain significance at the coarse smoothness level of 15 mm FWMH (see supplementary material).

## Discussion

This study is the first in the area of BFRBs to examine whether the clinical subtypes of pulling and picking have distinct neurobiological profiles. Partially supportive of our hypotheses, we found that two subtypes of people with TTM had distinct structural findings on MRI. First, low awareness hair pullers demonstrated increased cortical volume in the lateral occipital lobe. Low awareness hair pullers appear to be the most common subtype of TTM and are best characterized as having more automatic hair pulling, and more pulling due to emotional triggers. In addition to visual perception, the lateral occipital cortex is involved in touch- and proprioception-related sensory activation.

The other key finding was that our data demonstrate that impulsive/perfectionist hair pullers showed relatively decreased volume near the lingual gyrus of the inferior occipital-parietal lobe compared to controls and relative to sensory sensitive pullers. Research consistently shows that the lingual gyrus is associated with visual memory and motion imagery. Additionally, there is some indication that the lingual gyrus plays a role in response inhibition in the context of negative emotions^26^ and in reward processing.^27^ Adults with TTM have demonstrated deficits in response inhibition^28^ compared to individuals with no family history of BFRBs, and have exhibited dysfunctional reward processing as well.^29^ The fact that certain findings such as response inhibition have been somewhat mixed in TTM^30^ may speak to the fact that earlier studies were unaware of subtypes and that the impulsive/perfectionist subtype may be more likely to demonstrate these deficits.

Taken together, what do we make of these findings? These data are important because they show, albeit in small ways, that the clinical heterogeneity previously found in TTM may have partially distinct biological underpinnings. These particular volumetric differences may however only be part of the larger picture as it is doubtful that size of any single area tells the complete picture. In fact, we have yet to examine other factors such as white matter integrity and functional connectivity and so this is likely the first finding of a yet to be determined complex understanding of the clinical heterogeneity of TTM.

It is also important to note non-significant results herein. We did not find that one subtype referred to as “sensory sensitive pullers,” differed from the other clinical subtypes or controls in any structural manner. Similarly, the clinical phenotyping of skin picking disorder did not show significant differences in brain structure in those subtypes either. The lack of structural differences in these other subtypes leads to several possible explanations: perhaps these subtypes are less meaningful biologically. In fact, there was some suggestion based on the clinical phenotyping data that perhaps the subtypes of skin picking were actually reflective of symptom severity differences.^21^ If so, then neuroimaging of structural biology may be less useful in understanding the picking subtypes. Another explanation is that certain subtypes may have more subtle biological differences and thereby require a larger sample or a different imaging modality.

Several limitations should be considered in relation to the current study. Although the total size of the study was fairly large, the number of participants with individual

BFRBs may have been too small to detect differences between the subtypes. In addition, the sample was not large enough to examine the role of comorbidities. Finally, the current research was undertaken in a cohort that was largely female and of white racial-ethnic type and thus may not be representative of the larger population of people with BFRBs.

In summary, this is the first neuroimaging study to explore data-driven subtypes of BFRBs. We found evidence of some volumetric brain differences between specific hair pulling subtypes, which were robust with stringent statistical correction. Future work should further explore neurobiological underpinnings of BFRB subtypes using a range of imaging modalities, and explore whether the subtypes show differential response to treatments – both in terms of changes in symptoms and brain structure/function.

## Supplementary Material

Supplementary materials

## Figures and Tables

**Figure 3:**
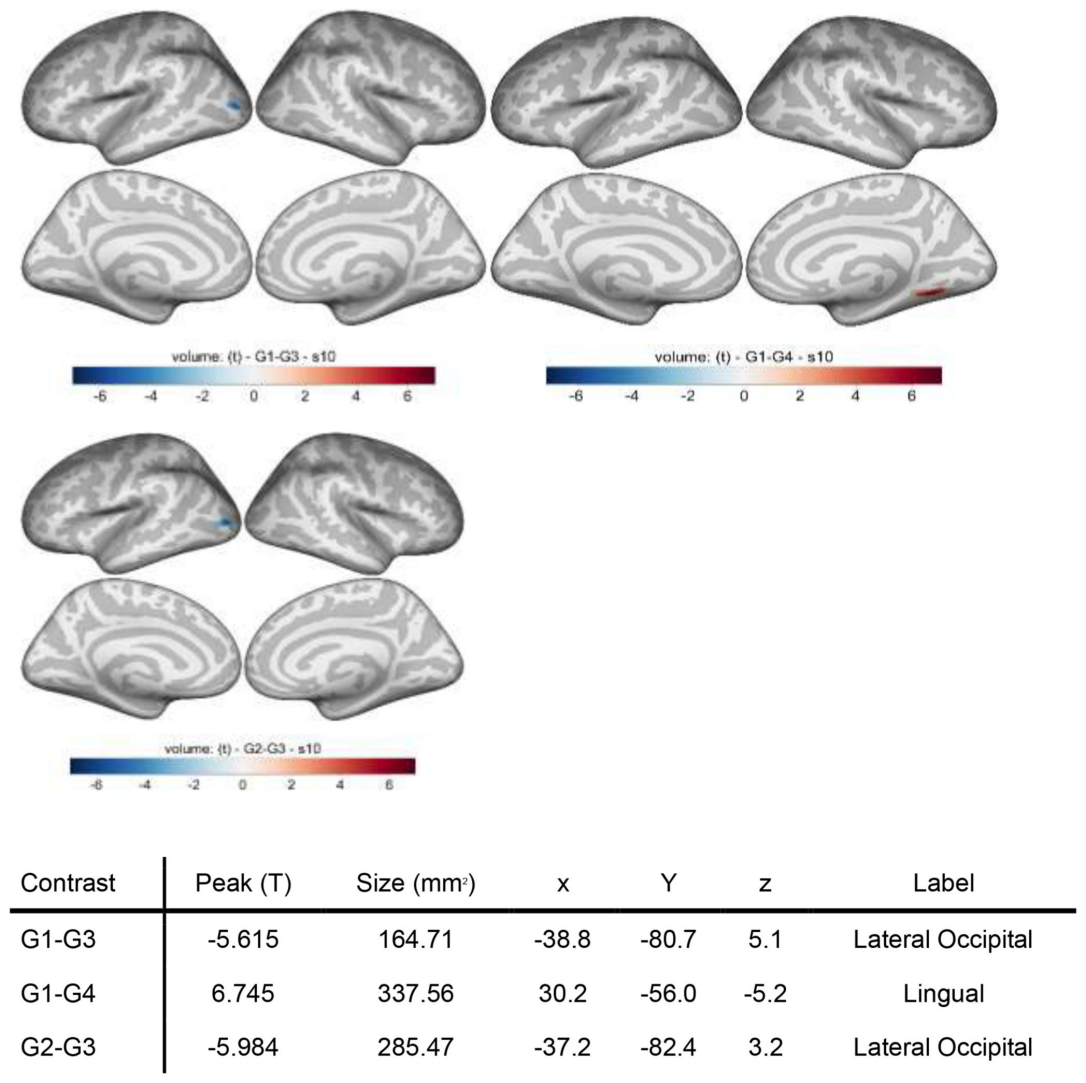
Cortical volume differences across hair pulling subtypes

**Table 1:** Clinical subtypes of pulling and picking participants based on mixture modeling (Grant et al., 2020)

Puller Type	Sex	N	age	min_age	max_age	sexRatio
Controls (i.e. subtype 1)	Female	93	31	18	68	84.55
	Male	17	27.7	21	36	15.45
Subtype 2	Female	18	34.6	18	57	78.26
	Male	5	29.4	18	36	21.74
Subtype 3	Female	58	28.1	19	56	86.57
	Male	9	27	18	41	13.43
Subtype 4	Female	31	29.5	20	56	77.5
	Male	9	27.2	19	36	22.5

Picker Type	Sex	N	age	min_age	max_age	sexRatio
Controls (i.e. subtype 1)	Female	92	30.4	19	63	87.62
	Male	13	27.4	18	38	12.38
Subtype 2	Female	80	29.6	18	59	80.81
	Male	19	27.4	20	36	19.19
Subtype 3	Female	34	31.7	19	68	80.95
	Male	8	28.6	18	41	19.05

Puller subtypes: Subtype 2: Sensory sensitive pullers; Subtype 3: Low awareness pullers; Subtype 4: Impulsive/Perfectionist pullers

Picker subtypes: Subtype 2: Emotional/reward pickers; Subtype 3: Functional pickers
