# Supplementary material for: Neurobiology of Subtypes of Trichotillomania and Skin Picking Disorder
Source: CNS Spectr. Author manuscript; Available in PMC 2023 Feb 23. (PMC7614223; doi:10.1017/S109285292100095X)

## Supplementary materials

### Case-Control group comparisons across thresholds

#### Cortical thickness


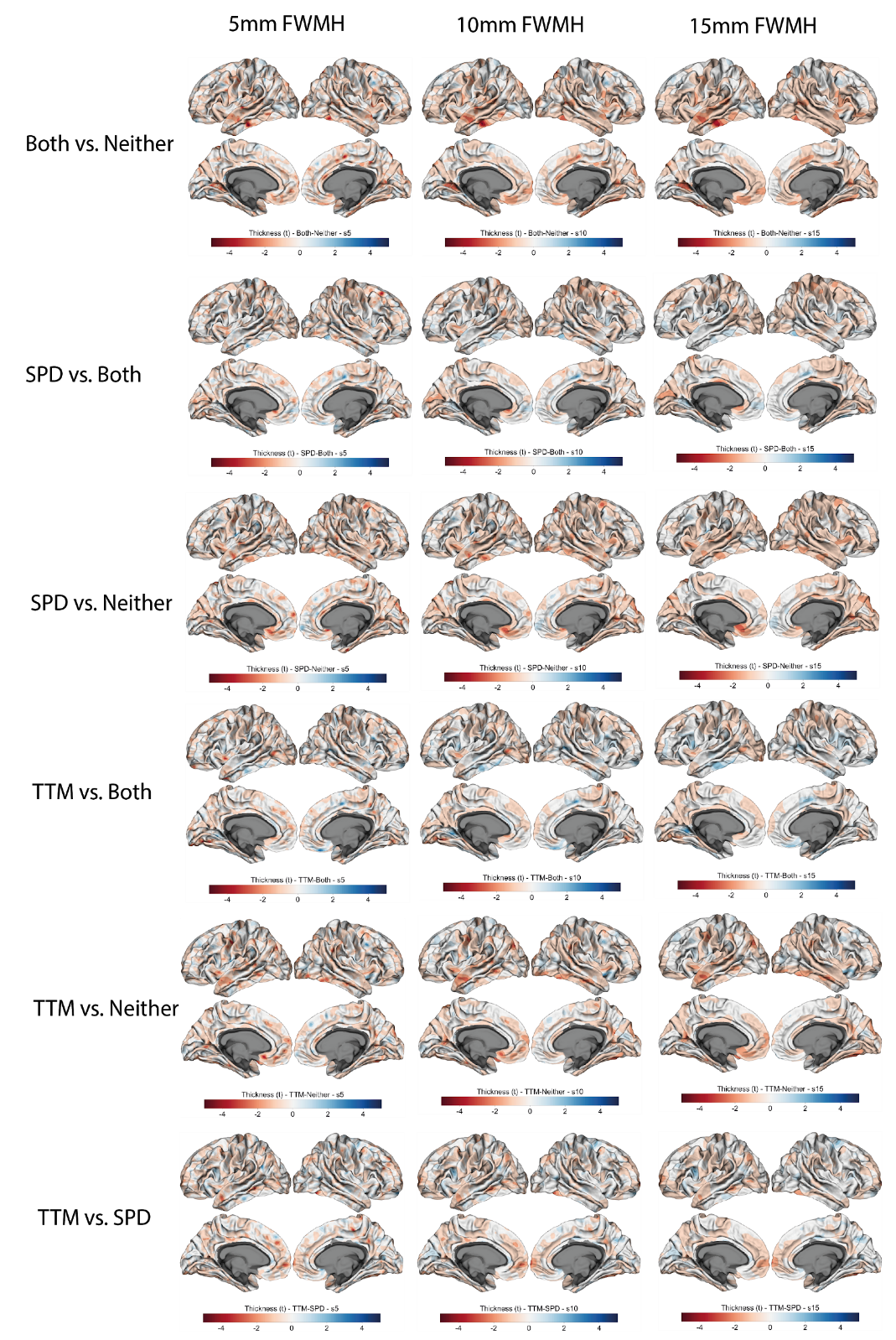


#### Surface area


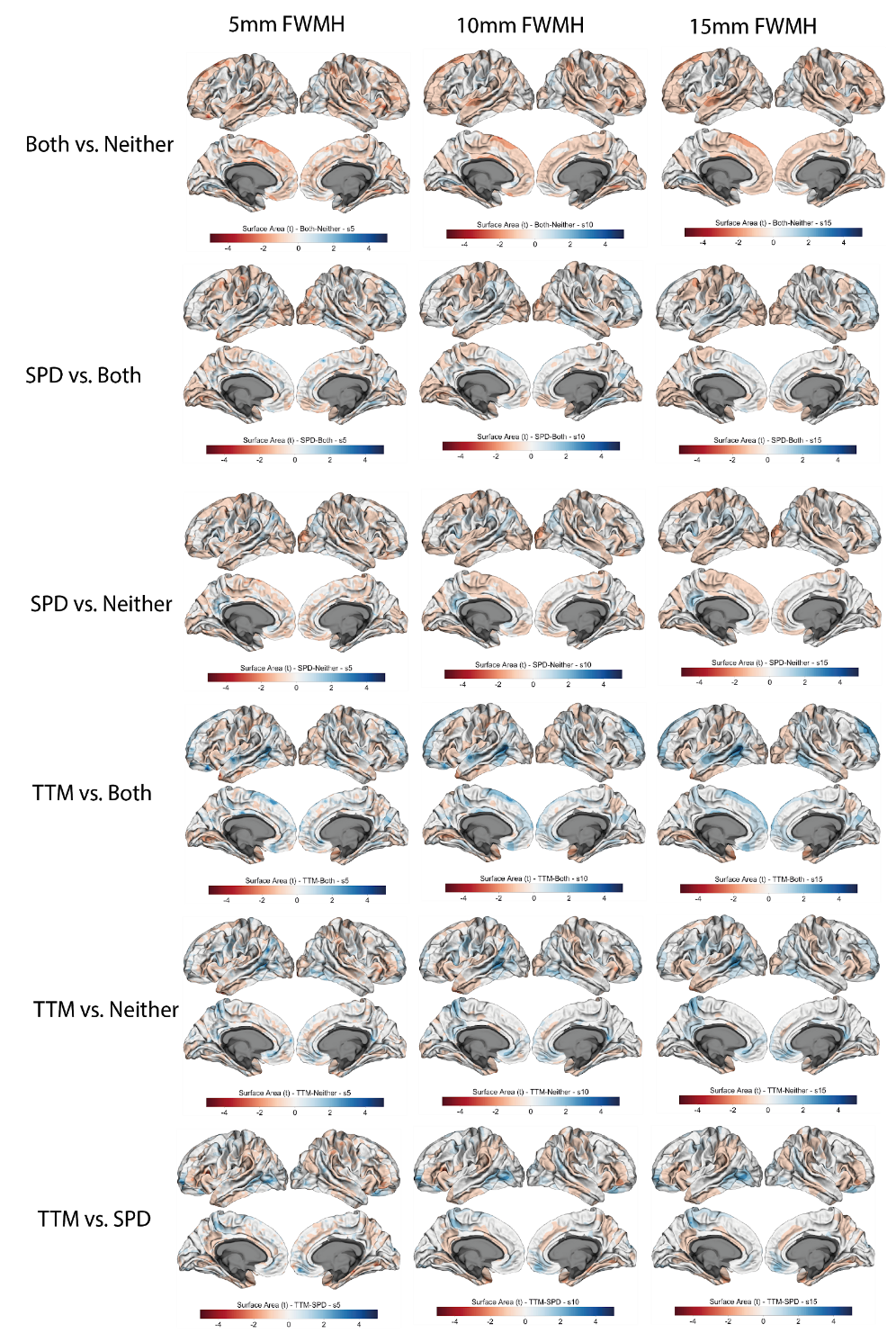


#### Volume


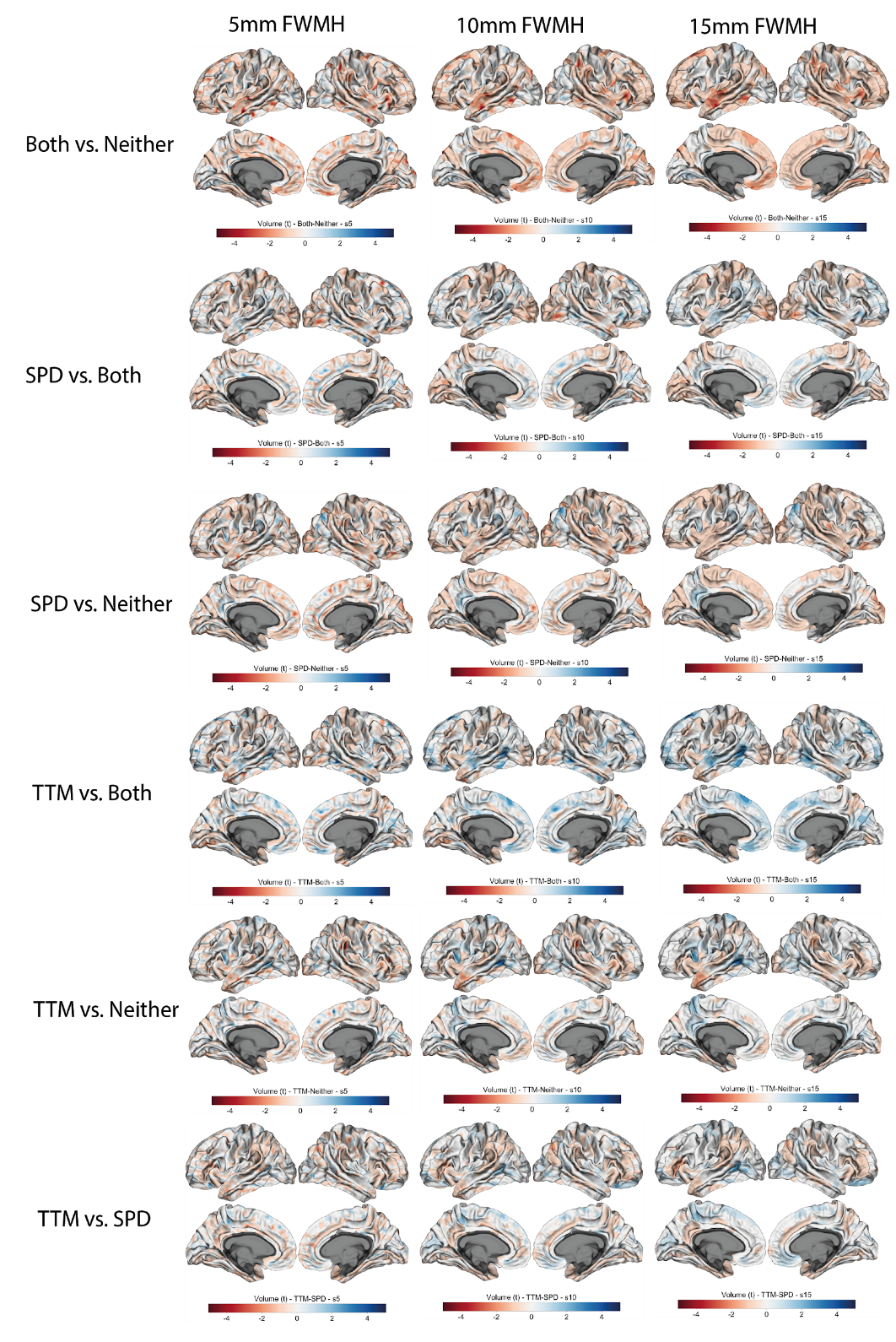


### Puller subtype significant differences across 2 smoothness kernels

In some cases there were significant differences that did not appear across the different levels of smoothness but did survive cluster-correction at a smoothness of 5 and 10mm FWMH. For completeness those are reported below.

#### Surface area


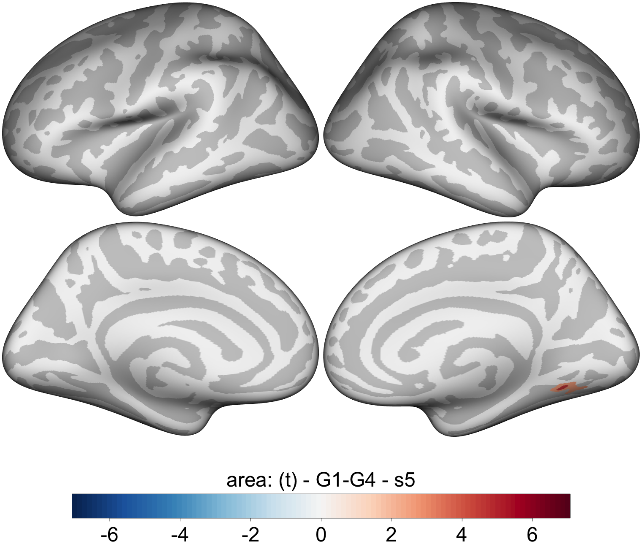

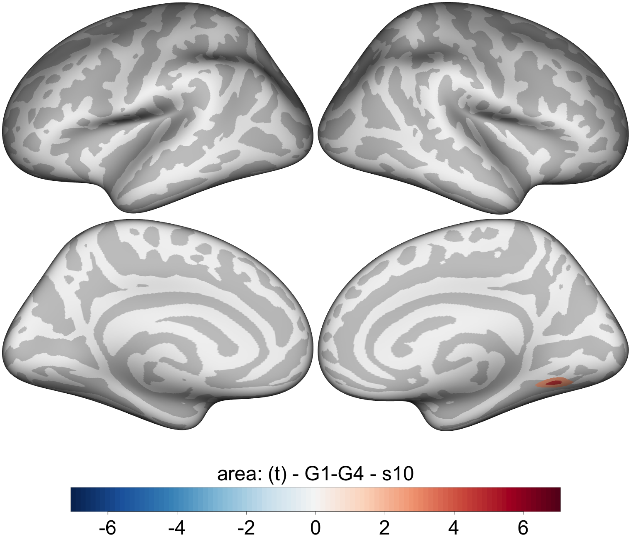


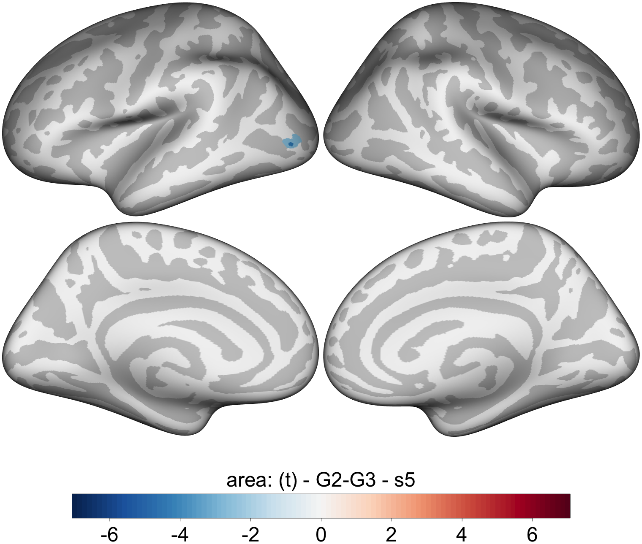

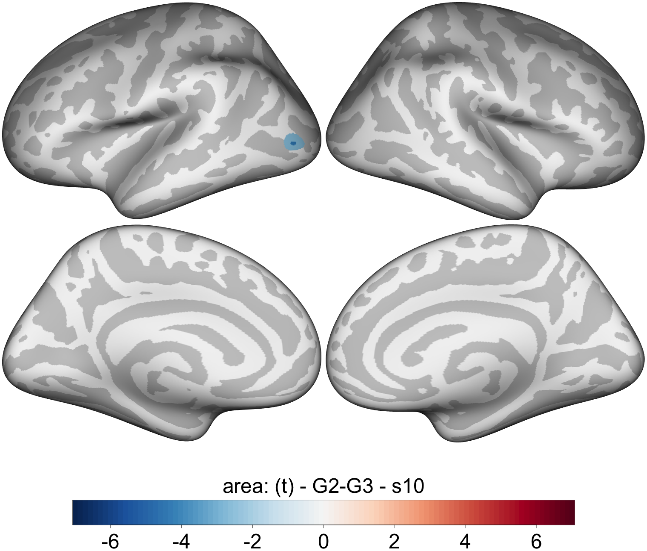


#### Volume


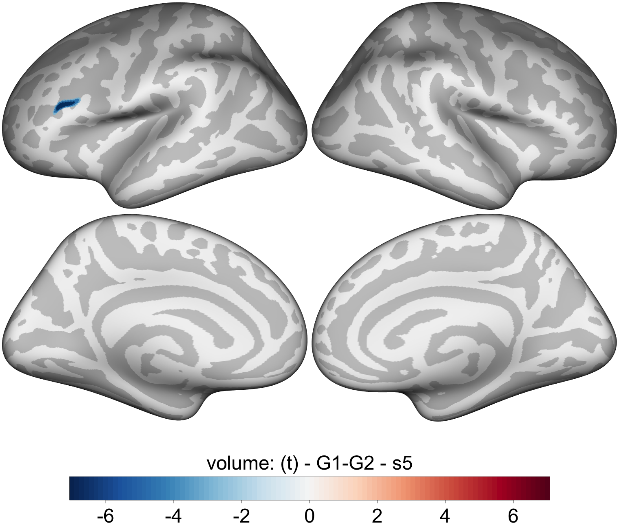

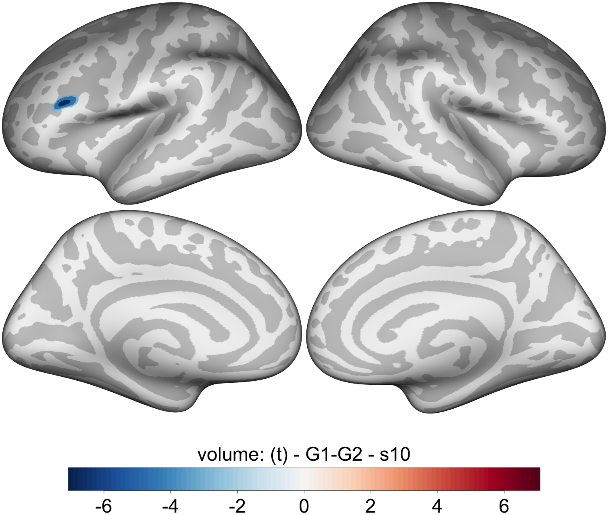


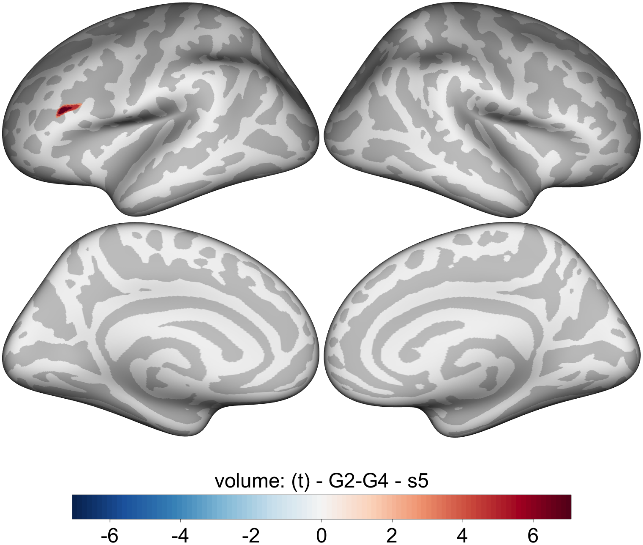

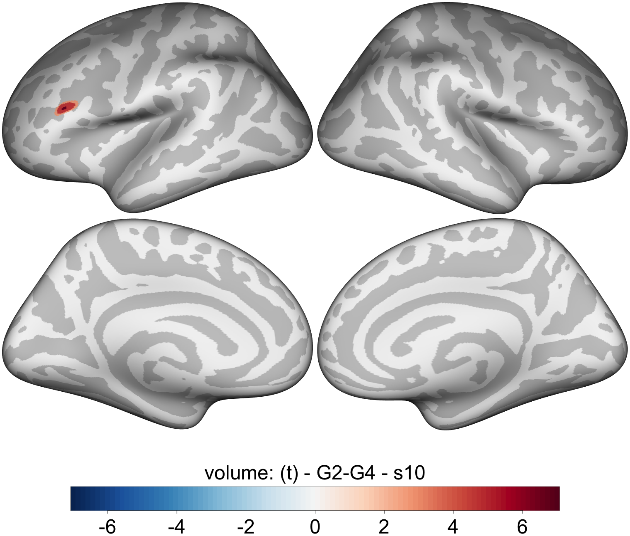


####

### Picker subtype significant differences across 2 smoothness kernels

In some cases there were significant differences that did not appear across the different levels of smoothness but did survive cluster-correction at a smoothness of 5 and 10mm FWMH. For completeness those are reported below.

#### Volume


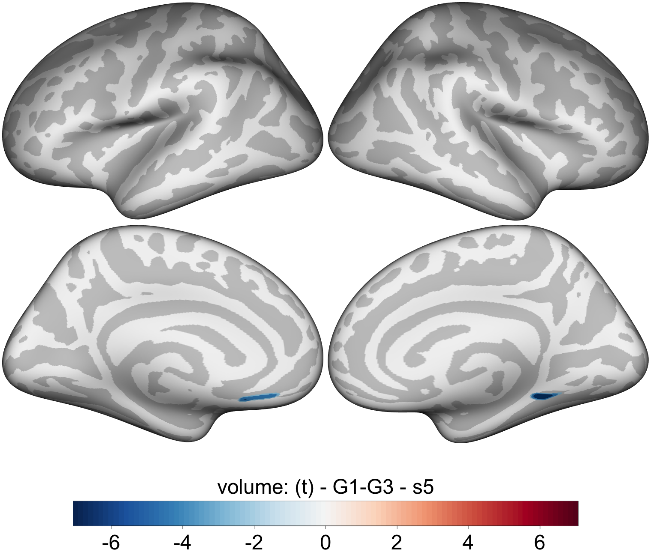

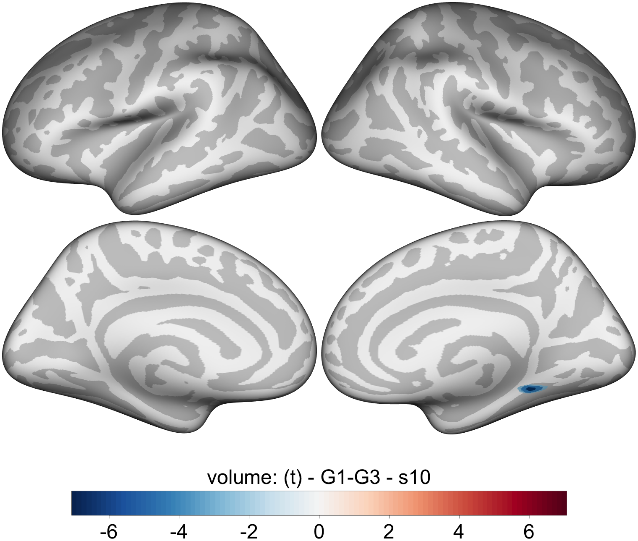

Supplement: Supplementary materials [file EMS140610-supplement-Supplementary_materials.docx]
